# Self-Injury Among Left-Behind Adolescents in Rural China: The Role of Parental Migration and Parent–Child Attachment

**DOI:** 10.3389/fpsyg.2018.02672

**Published:** 2019-01-07

**Authors:** Yulong Wang, Manqi Zhang, Huiling Chen

**Affiliations:** ^1^School of Educational Science, Cognition and Human Behavior Key Laboratory of Hunan Province, Hunan Normal University, Hunan, China; ^2^Institute of Mental Health, Second Xiangya Hospital of Central South University, Changsha, China

**Keywords:** self-injury, parental migration, mother–child attachment, father–child attachment, left-behind adolescent

## Abstract

Previous studies have indicated that self-injury is a maladaptive coping strategy with a high prevalence among left-behind adolescents in rural China. However, few studies have been conducted on the factors influencing left-behind adolescents’ self-injury. The current study explored the roles of parental migration and parent–child attachment on self-injury. In total, 1110 adolescents were selected from four rural middle schools in Hunan province. Data on demographic and left-behind characteristics were collected and the Adolescent Self-Harm Scale and Inventory of Parent and Peer Attachment were administered. The results indicated that compared with non-left-behind children and children with one parent migrating, there was a higher prevalence of self-injury among children with two parents migrating. Those with lower levels of parent–child attachment had a higher prevalence of self-injury than those with higher levels of parent–child attachment. There were also significant differences in terms of frequency, severity, and overall level of self-injurious behavior by patterns of parental migration and levels of parent–child attachment. Thus, parental migration, parent–child attachment, and the interaction between parental migration and mother–child attachment can predict adolescents’ self-injury.

## Introduction

In China, a large surplus of the agricultural labor force has gradually been transferred to urban areas since the policies of reform and opening to the outside in the 1980s. Many rural parents who choose to make a living in the city must leave their children at home for social and economic reasons (Jiang, 2007), which brings about the phenomenon of left-behind children. In general, left-behind children are children under the age of 18 who do not live with their parents because one or both have moved away from the child’s household registration and living area (Duan and Zhou, 2005). The total number of left-behind children in rural China reached 40.51 million in 2015, among which the number of left-behind children aged between 12 and 17 years was 10.61 million, accounting for 26.19% of those left behind. Although the total number of left-behind children demonstrated an overall downward trend since 2010, it was still very large in absolute numbers as of 2017 (Duan et al., 2017). Left-behind children receive less parental care and have less hope due to separation from their parents during their development; this puts them at a psychological disadvantage (Fan et al., 2016). Some studies indicate that left-behind children have more behavioral problems than non-left-behind children, including self-injury (Hou et al., 2014; Liao et al., 2015; Zhao and Yu, 2016; Wang et al., 2017).

Self-injury refers to an individual intentionally and repeatedly physically harming themselves without the intention of suicide; this behavior is often not accepted by society and is mainly manifested in the form of cuts and burns (Klonsky and Olino, 2008; Nock and Favazza, 2009). In general, the frequency or severity of self-injurious behavior is considered as a useful indicator of injury (Gratz, 2001; Prinstein, 2008), but Feng (2008) suggested that the overall level of self-injury was a comprehensive indicator that fully considered the frequency and severity of injury to the body, which more accurately reflects the self-injury state of adolescents. Many studies have found that self-injury is associated with numerous psychological disorders (Selby et al., 2012; Doksat et al., 2017) and predicts future suicidal intention and behavior (Guan et al., 2012; Klonsky et al., 2013).

Left-behind adolescents are at high risk for self-injury. According to a study of China’s Anhui province, among 1920 left-behind adolescents, the rate of adolescents who had self-injury behavior at least once was 47.3%, which was far higher than the rate of the non-left-behind adolescents (Xu et al., 2010). Xu and Ma (2013) indicated that the overall prevalence rate of left-behind adolescents’ self-injury behaviors was high and there was a tendency for repeated occurrence. However, although children with one or two parents migrating are all left-behind, the impact of distinct parent migration on individual psychology and behavior may vary. For example, some studies found that compared with children with two parents migrating, children with one parent migrating and non-left-behind children had better social adaptability (Fan et al., 2009; Su et al., 2013), while another study found that there was no significant difference in terms of mental health status between children with one parent migrating and children with two parents migrating (Liu et al., 2012). Therefore, it is necessary to further explore the impact of distinct parent migration on adolescents’ self-injury.

Related literature has found that parent–child attachment was an important factor in self-injury (Wang et al., 2009, 2013; Bureau et al., 2010). Bowlby’s attachment theory suggests that the attachment between parent and child established early in life could be internalized as the internal working model of a child (Bowlby, 1973). This model reflects a very stable cognitive/affective structure and forms the basis of an individual’s emotions and reactions in other situations (Crowell and Treboux, 1995), thus having a far-reaching influence on the social adaptation and emotional regulation ability of the individual. Only a few related studies have found that both father–child attachment and mother–child attachment are significantly correlated with left-behind adolescents’ self-injury behavior, and the higher the attachment security, the less overall level of the self-injury behavior, such that parent–child attachment could directly or indirectly predict self-injury behavior (Wu, 2016).

Previous studies have extensively examined left-behind adolescents’ self-injurious behavior and found that parental migration may be a risk factor for it, but these studies have mainly considered left-behind adolescents as a whole, rather than looking at the impact of different parental migration situations (e.g., one parent migrating or two parents migrating) on self-injury behaviors (Xu et al., 2010; Xu and Ma, 2013). Moreover, the effects of special family structures (such as single parent family or orphans) has not been controlled for while the researchers examined left-behind adolescents’ self-injurious behavior. Existing research also shows that parent–children attachment may be a protection factor of left-behind adolescents’ self-injurious behavior in rural China (Wu, 2016). However, few studies have explored whether the impact of parent–child attachment on self-injury behavior of left-behind children varies due to distinct parent migration. Thus, it is necessary to conduct further research about the role of parental migration and parent–children attachment on self-injury among left-behind adolescents in rural China.

In the present study, we aimed to investigate the effect of parent–child attachment on the relationship between different parent migration situations and self-injury of left-behind adolescents. This study attempted to answer the following questions: (1) What are the demographic characteristics of left-behind adolescents with two parents migrating, left-behind adolescents with one parent migrating, and non-left-behind adolescents? (2) Is there any difference in the prevalence, frequency, severity, and overall level of self-injury among adolescents with different parent migrations? (3) Is there any difference in the prevalence, frequency, severity, and overall level of self-injury among adolescents with distinct parent–child attachment? (4) What is the effect of parent–child attachment on self-injury of adolescents with different parent migrations?

## Materials and Methods

### Study Population and Procedures

Samples were drawn from four rural schools belonging to three different areas of Hunan province and included left-behind adolescents and non-left-behind adolescents (control group). Hunan province is located in central China, which is one of the largest labor export provinces. In Hunan province, the number of left-behind children reached 2.73 million in 2015, accounting for 6.75% of the national rural left-behind children and ranking fourth in China (Duan et al., 2017).

The sample selection procedures were as follows: (1) We contacted four rural secondary schools (two high schools and two junior high schools) and obtained permission to conduct a questionnaire survey; (2) Six psychology graduate students completed training on the research design, instructions for inviting students to participate in the survey, and procedure for obtaining informed consent of students in the classrooms; and (3) Two classes were randomly selected from each grade of each school, with 24 classes in total. The selection criteria of left-behind adolescents were: (1) from rural areas; (2) one or both parents living far from home as migrant workers for 6 months or longer at a time; and (3) supervised by one parent, grandparents, or others. As the control group, non-left-behind adolescents were also selected from the same schools.

A total of 1285 questionnaires were handed out. After excluding questionnaires from orphans and adolescents from single-parent families (*n* = 87) and those with missing data (*n* = 88), 1110 valid questionnaires were collected.

### Measurements

#### Demographic Characteristics

All participants were asked to provide their gender, age, grade, and parents’ education levels (primary school or lower, secondary school or polytechnic school, university or higher [including junior college]).

#### Characteristics of Left-Behind Adolescents

The left-behind adolescents were required to answer several questions regarding their experience with parental migration. Sample questions included “How long has it been since your father/mother migrated?” (1 = less than a year, 2 = 1 to 3 years, 3 = 3 to 5 years, 4 = more than 5 years), “How old were you when your father/mother migrated?” (1 = younger than 3 years old, 2 = 3 to 6 years old, 3 = 6 to 12 years old, 4 = older than 12 years old), and “Who is your primary caregiver?” (1 = parents, 2 = father, 3 = mother, 4 = grandparents, 5 = other).

#### Self-Injury

The Adolescent Self-Harm Scale was revised by Feng (2008) based on the existing scale consisting of 18 items and 1 short answer question to assess frequency, severity, and overall level of self-injurious behavior. In this study, since the short answer question was not completed, only the results of the 18 items were counted. The items for frequency of self-injurious behavior were rated on a 4-point scale (0 = never, 1 = once, 2 = twice to four times, 3 = five times or more) and the items for severity of self-injurious behavior were rated on a 5-point scale (0 = none, 1 = mild, 2 = moderate, 3 = severe, 4 = extremely severe). The overall level of self-injurious behavior was evaluated as the product of the frequency and severity of self-injurious behavior. Whether the overall level of self-injurious behavior was 0 or greater was the criterion used to judge the absence or presence of self-injurious behavior. In the present study, the indicator of self-injury includes frequency, severity, and overall level. The original scale had good validity and Cronbach’s α was 0.85. To assess the time of self-injurious behavior accurately, the time of occurrence of self-injury was limited to less than 1 year. In the current study, Cronbach’s α was 0.91.

#### Parent–Child Attachment

The original version of the Inventory of Parent and Peer Attachment (IPPA) based on Bowlby’s attachment theory was developed by Armsden and Greenberg (1987), including three subscales (each with 25 items); it has been widely employed to assess adolescents’ parent–child attachment and peer attachment. The items are rated on a 5-point Likert scale ranging from 1 (not at all) to 5 (always true). Each subscale has three dimensions: trust (understanding and respect), communication (communication mode), and alienation (individual anger and neglect). The alienation subscale adopted reverse scoring. The current study employed the revised version of the IPPA by Lu (2006), which deleted items that had correlations with the total score less than 0.03: Items 9, 14, and 23 (father–child attachment); and Items 34, 39, and 48 (mother–child attachment). Items 3, 20, and 21 (father–child attachment) and Items 45 and 46 (mother–child attachment) were deleted after exploratory factor analysis. Both reliability and validity of the revised version were shown to be good. In the current study, Cronbach’s α of father–child attachment was 0.91 and that of mother–child attachment was 0.94.

### Data Analysis

First, a chi-squared test (for categorical variables) or analysis of variance (ANOVA; for continuous variables) was employed to examine differences in individual demographic characteristics among the three groups of rural adolescents. Second, a chi-square test was used to explore differences in life experiences related to parental migration between the two groups of left-behind adolescents. Third, a chi-square test was performed to explore differences in self-injurious behaviors by pattern of parental migration and overall level of parent–child attachment. Fourth, ANOVA was used to compare group differences in terms of frequency, severity, and overall level of self-injurious behavior by patterns of parental migration and levels of parent–child attachment. Finally, a general linear model (GLM) was performed to simultaneously assess group differences in self-injurious behavior by patterns of parental migration, parent–child attachment, and their interaction.

### Ethics Statement

The study was reviewed and approved by the IRB, Institute of Psychology, Hunan Normal University, and carried out in accordance with the recommendations, with written informed consent from all participants’ guardians. All participants’ guardians gave written informed consent in accordance with the Declaration of Helsinki.

## Results

### Sample Characteristics

The characteristics of the participants are shown in Table 1. Among the 1110 participants, 204 (18.4%) were adolescents with two parents migrating, 356 (32.1%) were adolescents with one parent migrating, and 653 (56.6%) were comparison adolescents. There were no significant differences between the male-to-female ratios, age, and education levels among the three groups. Both fathers’ and mothers’ education levels differed among the three groups, and differences reached statistical significance. The proportion of fathers with college or higher education was only 16% among adolescents with two parents migrating, while the proportions in adolescents with one parent migrating and comparison adolescents were both more than 20%. The proportion of mothers with college or higher education was less than 10% among adolescents with two parents migrating, while the proportions in adolescents with one parent migrating and comparison adolescents were 18.1 and 23.1%.

**Table 1:** Demographic characteristics of the sample.

	Overall	Children with two parents migrating	Children with one parent migrating	Comparison children	*F*/*χ*^2^	*P*-value
*n*	1110 (100%)	204 (18.4%)	356 (32.1%)	550 (49.5%)	162.57	<0.001
Gender					0.26	0.88
Male	556 (50.1%)	100 (49.0%)	182 (51.1%)	274 (49.8%)		
Female	554 (49.9%)	104 (51.0%)	174 (48.9%)	276 (50.2%)		
Age (M ± SD)	14.36 ± 1.81	14.40 ± 1.73	14.42 ± 1.66	14.32 ± 1.66	0.36	0.70
Educational level					3.58	0.17
Junior middle school	488 (44.0%)	101 (49.5%)	157 (44.1%)	230 (41.8%)		
Middle school	622 (56.0%)	103 (50.5%)	199 (55.9%)	320 (58.2%)		
Father’s educational level					15.53	<0.01
Primary school or lower	134 (12.3%)	32 (16.0%)	38 (10.8%)	64 (11.8%)		
Middle school	689 (62.9%)	136 (68.0%)	230 (65.5%)	321 (59.2%)		
College or higher	272 (24.9%)	32 (16.0%)	83 (23.6%)	157 (29.0%)		
Mother’s educational level					24.54	<0.001
Primary school or lower	175 (16.0%)	45 (22.6%)	56 (15.9%)	74 (13.7%)		
Middle school	712 (65.1%)	137 (68.8%)	233 (66.0%)	342 (63.2%)		
College or higher	206 (18.8%)	17 (8.5%)	64 (18.1%)	125 (23.1%)		

### Characteristics of Left-Behind Adolescents

As Table 2 illustrates, 61% of adolescents with two parents migrating reported being younger than 6 years old at separation; however, 41% of adolescents with one parent migrating reported the same experience. Generally, compared with adolescents with one parent migrating, adolescents with two parents migrating reported a longer duration of father migration (i.e., more than 6 years). The primary caregivers for most adolescents with two parents migrating (70.4%) were grandparents, while the primary caregivers for most adolescents with one parent migrating (60.5%) were mothers.

**Table 2:** Characteristics of left-behind children.

	Children with two parents migrating	Children with one parent migrating	*χ*^2^	*P*-value
Sample size	204 (36.4%)	356 (63.6%)	41.26	<0.001
Age when father migrated (years old)			11.73	<0.01
≤3	75 (36.9%)	94 (29.2%)		
3–6	49 (24.1%)	54 (16.8%)		
6–12	51 (25.1%)	115 (35.7%)		
≥12	28 (13.8%)	59 (18.3%)		
Age when mother migrated (years old)			1.53	0.68
≤3	66 (33.8%)	45 (33.8%)		
3–6	44 (22.6%)	33 (42.9%)		
6–12	54 (27.7%)	40 (30.1%)		
≥12	31 (15.9%)	15 (11.3%)		
Length of paternal migration (years)			10.95	<0.05
≤1	62 (31.2%)	132 (41.8%)		
1–3	30 (15.1%)	55 (17.4%)		
3–5	15 (7.5%)	28 (8.9%)		
≥5	92 (46.2%)	101 (32.0%)		
Length of maternal migration (years)			7.59	0.06
≤1	62 (31.8%)	51 (39.2%)		
1–3	37 (19.0%)	31 (23.8%)		
3–5	21 (10.8%)	17 (13.1%)		
≥5	75 (38.5%)	31 (23.8%)		
Primary caregiver			351.37	<0.001
Father		25 (9.5%)		
Mother		159 (60.5%)		
Grandfather/ grandmother	100 (70.4%)	54 (20.5%)		
Other	42 (29.6%)	25 (9.5%)		

### Relationship of Self-Injury With Parental Migration

Table 3 demonstrates the group differences in terms of prevalence, frequency, severity, and overall level of self-injurious behavior by patterns of parental migration. The patterns of parental migration (i.e., two parents migrating, one parent migrating, or no parents migrating) were found to be significantly associated with prevalence, frequency, severity, and overall level of self-injurious behavior. The *post hoc* comparison revealed that adolescents with two parents migrating reported higher scores on frequency of self-injurious behavior than adolescents with no parents migrating, and higher prevalence and higher scores on severity and overall level of self-injurious behavior than comparison adolescents or adolescents with one parent migrating.

**Table 3:** Differences in prevalence, frequency, severity, and overall level of self-injurious behavior among parental migration groups.

	Prevalence of self-injury	Frequency of self-injury	Severity of self-injury	Overall Level of self-injury
Children with two parents migrating (1)	43.6%	10.0 ± 9.22	4.75 ± 5.73	9.87 ± 13.56
Children with one parent migrating (2)	30.6%	7.95 ± 6.46	3.51 ± 3.44	7.11 ± 8.03
Comparison children (3)	30.7%	7.66 ± 7.08	3.23 ± 3.27	6.47 ± 7.86
*F*/*χ*^2^	12.61^∗∗^	3.02^∗^	4.28^∗^	3.77^∗^
*Post hoc* comparison	1 > 2, 3	1 > 3	1 > 2, 3	1 > 2, 3

*^∗^p < 0.05, ^∗∗^p < 0.01.*

### Relationship of Self-Injury With Parent–Child Attachment

Table 4 shows that father–child attachment demonstrated a significant effect on prevalence, frequency, severity, and overall level of self-injurious behavior. The *post hoc* comparison indicated that adolescents with high or middle levels of father–child attachment reported lower prevalence and lower scores on frequency, severity, and overall level of self-injurious behavior than adolescents with low levels of father–child attachment. Mother–child attachment demonstrated a significant effect on prevalence, frequency, severity, and overall level of self-injurious behavior. The *post hoc* comparison indicated that adolescents with high or middle levels of mother–child attachment reported lower prevalence and lower scores on frequency and severity of self-injurious behavior than adolescents with low levels of mother–child attachment. In addition, adolescents with high levels of mother–child attachment reported lower scores on overall level of self-injurious behavior than adolescents with low levels of mother–child attachment.

**Table 4:** Differences in prevalence, frequency, severity, and overall level of self-injurious behavior among parent–child attachment groups.

	Prevalence of self-injury	Frequency of self-injury	Severity of self-injury	Overall level of self-injury
**Father–child attachment (M, SD)**				
Higher level (1)	22.7%	6.25 ± 5.84	2.92 ± 4.18	4.97 ± 6.71
Middle level (2)	32.4%	8.01 ± 6.88	3.40 ± 3.35	6.91 ± 8.17
Lower level (3)	47.2%	11.11 ± 9.93	5.30 ± 5.67	11.61 ± 14.39
*F*/χ^2^	25.66^∗∗∗^	7.43^∗∗^	7.29^∗∗^	9.08^∗∗∗^
*Post hoc* comparison	1 < 2 < 3	1, 2 < 3	1, 2 < 3	1, 2 < 3
**Mother–child attachment (M, SD)**				
Higher level	22.6%	6.97 ± 6.84	3.01 ± 3.83	5.41 ± 7.64
Middle level	31.4%	8.21 ± 7.39	3.54 ± 3.67	7.17 ± 8.60
Lower level	50.5%	10.32 ± 8.55	5.08 ± 5.56	11.21 ± 14.23
*F*/χ^2^	36.27^∗∗∗^	3.14^∗^	4.44^∗^	6.04^∗∗^
*Post hoc* comparison	1, 2 < 3; 1 < 2	1 < 3	1 < 3	1, 2 < 3

*^∗^p < 0.05, ^∗∗^p < 0.01, ^∗∗∗^p < 0.001.*

### Effect of Parental Migration and Parent–Child Attachment on Self-Injury of Left-Behind Adolescents

After controlling for two key individual characteristics (age and gender), GLM analysis of frequency, severity, and overall level of self-injurious behavior was employed to explore the effect of the pattern of parental migration and parent–child attachment on self-injurious behavior among adolescents.

Table 5 illustrates that the patterns of parental migration, father–child attachment, and mother–child attachment had main effects in terms of frequency, severity, and overall level of self-injurious behavior after the key individual characteristics were controlled. A significant interaction was found between patterns of parental migration and mother–child attachment in the multivariate test. The results indicated that the effect of mother–child attachment on self-injury behavior was different with different patterns of parental migration, and greatest with parental monitoring (no parent migrating).

**Table 5:** General linear model analysis of frequency, severity, and overall level of self-injurious behavior (β).

Self-injurious behavior	Main effect			Interaction		Covariates	
			
	Patterns of parental migration	Father–child attachment	Mother–child attachment	Patterns of parental migration × father–child attachment	Patterns of parental migration × mother–child attachment	Age	Gender
Frequency	-0.12^∗^	-0.18^∗∗^	-0.21^∗∗∗^	-0.004	-0.09	0.004	-0.01
Severity	-0.14^∗∗^	-0.20^∗∗∗^	-0.19^∗∗∗^	0.07	0.04	-0.07	-0.01
Overall level	-0.13^∗^	-0.24^∗∗∗^	-0.21^∗∗∗^	0.05	0.11^∗^	-0.05	-0.03

*^∗^p < 0.05, ^∗∗^p < 0.01, ^∗∗∗^p < 0.001.*

## Discussion

This study compared the fundamental state of left-behind adolescents. The results indicated that compared with adolescents with two parents migrating, adolescents with one parent migrating had parents with higher education, an older age (older than 6 years old) when parents left, and shorter period of time that the father has worked away from home. The primary caregivers of most adolescents with two parents migrating were grandparents, while for adolescents with one parent migrating, the primary caregiver was usually the mother. The differences between the two groups suggest that adolescents with two parents migrating are living in less favorable conditions. For example, the lower level of education of parents indicates lower family socio-economic status (Degarmo et al., 1999), negatively affecting the mental health development of adolescents (Bradley and Corwyn, 2002). The duration of early parent–child separation has a direct negative predictive effect on parent–child attachment (Peng et al., 2017). In addition, fathers’ absence in childhood is a key factor affecting children’s interpersonal relationships and subjective well-being (Flouri and Buchanan, 2003). Even rearing provided by grandparents with sufficient education, parenting methods, and emotional support cannot equal that provided by parents (Edwards and Daire, 2006; Guo, 2014).

The present study found that there were significant differences in the prevalence of self-injury among adolescents with one and two parents migrating and comparison adolescents, such that the prevalence of self-injury of adolescents with two parents migrating was higher than that of the other two groups. Among the self-injured adolescents, adolescents with two parents migrating had a higher frequency and severity of self-injurious behavior than both adolescents with one parent migrating and comparison adolescents. The results suggest a relationship between the possibility of self-injury and aspects of behavior (frequency, severity, and overall level) and left-behind state, with adolescents with two parents migrating displaying more serious problems than adolescents with one parent migrating. In fact, although there have been several previous studies with similar findings (Fan et al., 2009; Su et al., 2013), very few paid close attention to causes of the differences in self-injury among left-behind adolescents. In terms of self-injurious behavior, the phenomenon that adolescents with two parents migrating have more problems is likely to be related to the fact that the primary caregiver of most adolescents with one parent migrating (60.5%) is the mother. There is a typical division of labor in the traditional Chinese family, which is referred to as “Men outside, women inside.” “Men outside” refers to fathers providing financial support for families and “women inside” means that the mother is responsible for raising children and providing emotional support for the whole family. Today, this typical division of labor has collapsed in urban families with social modernization. However, in rural areas, this pattern has been largely preserved. In a rural family, if only one person is required to go out to work, the person is often the father, while the mother usually stays at home. This means that for a rural family, whether in terms of social culture or personal opinion, it is acceptable for fathers to go out to work. At the same time, “severe father and kind mother” describes differences in how parents treat their children, which emerges from the division of labor in China (Sun and Lin, 2018). Thus, Chinese children prefer to communicate with their mother rather than their father. Since self-injury is a maladaptive emotional coping strategy (Messer and Fremouw, 2008), this suggests that talking with their parents might be helpful for these children. For adolescents with one parent migrating, they can talk to their remaining parent about their thoughts and feelings, and especially tend to do so if the remaining parent is their mother. However, for adolescents with two parents migrating, it is more difficult to share their feelings, due to the lack of face-to-face contact with their parents.

This study found that there were significant differences in the prevalence of self-injury among adolescents with different parent–child attachment security. The prevalence of self-injury was lower in adolescents with higher parent–child attachment security. Among left-behind adolescents with self-injurious behaviors, there were significant differences in the frequency, severity, and overall level of self-injury of adolescents with different parent–child attachment security. These results indicate that parent–child attachment security is related to adolescent self-injury. The affect regulation model considers that individual self-injury is aimed at evoking emotion or expression, and managing strong negative emotion, so as to control personal emotion (Messer and Fremouw, 2008). Therefore, self-injury can reflect a deficit of individual affect regulation ability. In addition, parent–child attachment is closely related to emotion (Rawatlal et al., 2015; Sümer and Harma, 2015; Yildiz, 2016). Hence, parent–child attachment is also important in understanding the development of children’s affect regulation ability. Zimmermann (1999) found adaptive affect regulation was significantly positively correlated with secure attachment, which was significantly negatively correlated with avoidant and ambivalent attachment. Further, Wang et al. (2016) found that parent–child attachment security could positively predict affect regulation ability of left-behind children. Thus, parent–child attachment security may be a predictive factor of adolescents’ self-injury.

Further analysis indicated that left-behind state, father–child attachment, and mother–child attachment had a significant predictive effect on the frequency, severity, and overall level of self-injury of adolescents, which is consistent with previous research (Xu et al., 2010; Wang et al., 2013; Claes et al., 2016), suggesting that parental migration (especially two parents migrating) is an important cause of self-injury behavior and secure parent–child attachment is an effective protective factor. In addition, the research shows an interaction of left-behind state and mother–child attachment on adolescents’ self-injury. This suggests that left-behind state and mother–child attachment work together to determine the overall level of adolescents’ self-injury behavior, and that no parents migrating and a high level of mother–child attachment offers the most effective protection against adolescents’ self-injury. As mentioned above, self-injury behavior is a typical manifestation of poor emotional health. Hence, the significant interaction means that parents who are present (especially the mother) and high mother–child attachment security play a key role in promoting emotional health for children. It is easier for children with two migrating parents to be ignored by their parents. An insecure attachment style develops as the result of early damaging caregiver-infant interactions, which is usually associated with lower levels of reflective functioning (RF) (Borelli et al., 2015). A recent study found that lower levels of RF could lead to an increased vulnerability to mental disorders in adolescence and adulthood, and subsequent lower levels of well-being (Borelli et al., 2018). Thus, it is possible that children in unfavorable situations with two parents migrating suffer from a combination of the lack of a mother role and low mother–child attachment security.

### Implications for Services

This study reveals the role of parental migration and parent–child attachment in adolescent self-injury behaviors, which can help to explore mechanisms of self-injury of left-behind adolescents in rural China. This study found that self-injury behaviors of adolescents with two parents migrating were more serious than those of adolescents with one parent migrating and comparison adolescents. This suggests that when it comes to left-behind adolescents, we should make more specific distinctions. In addition, it also shows that both parents migrating has a negative impact on adolescents’ mental health, and only one parent migrating may be a choice that minimizes the impact. According to data from China’s 1% population sampling survey in 2015, children with two parents migrating accounted for 48.09% of the left-behind children in rural areas (Duan et al., 2017). It is obviously unrealistic to eliminate left-behind children in the short term due to China’s developmental stage and basic national conditions; however, reducing the number of children with two parents migrating is possible. Therefore, the government and relevant social institutions should focus on reducing or even eliminating the group of children with two parents migrating. Another important result of this study is the role of parent–child attachment in self-injury behaviors of adolescents with different parental migration situations. Additionally, the interaction between mother–child attachment and parental migration is a noteworthy result. For the prevention and intervention of left-behind children’s related problems, parent–child attachment should be taken into consideration. For example, a more secure attachment should be established between parents and children before they leave their children for a long time, which means that parents should not leave when their child is of a young age. If it is necessary for parents to migrate in search of work to make a living, one of them should live at home to raise the children. In this study, the primary caregiver was the mother, which may be due to the traditional belief of “men outside, women inside” in rural China. However, the influence of the father on children’s growth cannot be denied. No matter who goes out to work, both mother and father should pay attention to communicating with their children to reduce the negative influence of absent parents.

### Limitations of This Study

There are some limitations of this research. First, the sample was made up of adolescents attending school, and did not include left-behind adolescents who dropped out of school or left school for other reasons. In fact, about 1.5% of left-behind adolescents between the ages of 12 and 14 and 2.47% of left-behind adolescents between the ages of 15 and 17 years old leave school (Duan et al., 2017), which may affect the representativeness of the sample. Second, all data in this study came from self-reports, which may lead to subjective biases. Finally, the cross-sectional design prevents a trajectory analysis of self-injury and a causal interpretation of the findings. In follow-up studies, sampling of left-behind youth groups at school and out of school should be considered. When collecting data, it is necessary to include adolescents and other insiders such as parents and teachers as much as possible, and to adopt a longitudinal study design to explore the dynamic relationship between independent variables and dependent variables.

## Author Contributions

YW: substantial contributions to the design of the work, data analysis, and writing. MZ: substantial contributions to the design of the work and writing. HC: substantial contributions to data collection.

## Conflict of Interest Statement

The authors declare that the research was conducted in the absence of any commercial or financial relationships that could be construed as a potential conflict of interest.

## References

[B1] Armsden G. C. Greenberg M. T. (1987). The inventory of parent and peer attachment: individual differences and their relationship to psychological well-being in adolescence. *J. Youth Adolesc.* 16 427–454. 10.1007/BF02202939 24277469

[B2] Borelli J. L. Brugnera A. Zarbo C. Rabboni M. Bondi E. Tasca G. A. (2018). Attachment comes of age: adolescents’ narrative coherence and reflective functioning predict well-being in emerging adulthood. *Attach. Hum. Dev.* 2008 1–20. 10.1080/14616734.2018.1479870 29865892

[B3] Borelli J. L. Compare A. Snavely J. E. Decio V. (2015). Reflective functioning moderates: the association between perceptions of parental neglect and attachment in adolescence. *Psychoanal. Psychol.* 32 23–35. 10.1037/a0037858

[B4] Bowlby J. (1973). *Attachment and Loss: Vol. 2. Separation: Anxiety and Anger*. New York, NY: Basic Books

[B5] Bradley R. H. Corwyn R. F. (2002). Socioeconomic status and child development. *Annu. Rev. Psychol.* 53 371–399. 10.1146/annurev.psych.53.100901.135233 11752490

[B6] Bureau J. F. Martin J. Freynet N. Poirier A. A. Lafontaine M. F. Cloutier P. (2010). Perceived dimensions of parenting and non-suicidal self-injury in young adults. *J. Youth Adolesc.* 39 484–494. 10.1007/s10964-009-9470-4 19882378

[B7] Claes L. Raedt R. D. Walle M. V. D. Bosmans G. (2016). Attentional bias moderates the link between attachment-related expectations and non-suicidal self-injury. *Cogn. Ther. Res.* 40 540–548. 10.1007/s10608-016-9761-5

[B8] Crowell J. A. Treboux D. (1995). A review of adult attachment measures: implications for theory and research. *Soc. Dev.* 4 294–327. 10.1111/j.1467-9507.1995.tb00067.x

[B9] Degarmo D. S. Forgatch M. S. Martinez C. R. Jr. (1999). Parenting of divorced mothers as a link between social status and boys’ academic outcomes: unpacking the effects of socioeconomic status. *Child Dev.* 70 1231–1245. 10.1111/1467-8624.00089 10546342

[B10] Doksat N. G. Zahmacioglu O. Demirci A. C. Kocaman G. M. Erdogan A. (2017). Association of suicide attempts and non-suicidal self-injury behaviors with substance use and family characteristics among children and adolescents seeking treatment for substance use disorder. *Subst. Use Misuse* 52 604–613. 10.1080/10826084.2016.1245745 28140729

[B11] Duan C. R. Lai M. H. Qin M. (2017). Research on the change trend of left-behind children in rural China since the 21st century. *China Youth Study* 6 52–60.

[B12] Duan C. R. Zhou F. L. (2005). A study on children left behind. *Popul. Res.* 29 29–36.

[B13] Edwards O. W. Daire A. P. (2006). School-age children raised by their grandparents: problems and solutions. *J. Instr. Psychol.* 33 113–119.

[B14] Fan X. H. Fang X. Y. Liu Q. X. Liu Y. (2009). A social adaptation comparison of migrant children, rear children, and ordinary children. *J. Beijing Normal Univ.* 215 33–40.

[B15] Fan X. H. He M. Chen F. J. (2016). Effect of hope on the relationship between parental care and loneliness in left-behind children in rural China. *Chin. J. Clin. Psychol.* 24 702–706.

[B16] Feng Y. (2008). *The Relation of Adolescents’ Self-Harm Behaviors, Individual Emotion Characteristics and Family Environment Factors*. Wuhan: Central China Normal University.

[B17] Flouri E. Buchanan A. (2003). The role of father involvement in children’s later mental health. *J. Adolesc.* 26 63–78. 10.1016/S0140-1971(02)00116-1 12550822

[B18] Gratz K. L. (2001). Measurement of deliberate self-harm: preliminary data on the deliberate self-harm inventory. *J. Psychopathol. Behav. Assess.* 23 253–263. 10.1023/A:1012779403943

[B19] Guan K. Fox K. R. Prinstein M. J. (2012). Nonsuicidal self-injury as a time-invariant predictor of adolescent suicide ideation and attempts in a diverse community sample. *J. Consult. Clin. Psychol.* 80 842–849. 10.1037/a0029429 22845782 PMC3458144

[B20] Guo X. L. (2014). The influence of grandparenting on cognitive development among children: a longitudinal study. *Chin. J. Clin. Psychol.* 22 1072–1077.

[B21] Hou K. Liu Y. Qu Z. Y. Jiang S. (2014). The social adjustment of left-behind children in rural China: a propensity score analysis. *Psychol. Dev. Educ.* 30 646–655.

[B22] Jiang Y. C. (2007). The family’s social capital and the “left-behind children” network of families– the national survey of Hunan lake village. *South China Popul.* 22 31–37.

[B23] Klonsky E. D. May A. M. Glenn C. R. (2013). The relationship between nonsuicidal self-injury and attempted suicide: converging evidence from four samples. *J. Abnorm. Psychol.* 122 231–237. 10.1037/a0030278 23067259

[B24] Klonsky E. D. Olino T. M. (2008). Identifying clinically distinct subgroups of self-injurers among young adults: a latent class analysis. *J. Consult. Clin. Psychol.* 76 22–27. 10.1037/0022-006X.76.1.22 18229979

[B25] Liao C. J. Wu J. X. Zhang J. F. (2015). A factor analysis of the mental health of children left behind: perspective of sense of security. *J. East China Normal Univ.* 33 88–97.

[B26] Liu X. H. Wang X. J. Yang Y. Y. Ha L. N. Li Q. L. Dai X. Y. (2012). Comparison of mental health status between left-behind children under different guardianships and non-left-behind children. *Chin. Gen. Pract.* 15 1507–1510.

[B27] Lu Y. (2006). *Relation Among Depressive Symptoms, Attachment and General Self-Efficacy of Junior Middle School Students*. Shijiazhuang: Hebei Normal University.

[B28] Messer J. M. Fremouw W. J. (2008). A critical review of explanatory models for self-mutilating behaviors in adolescents. *Clin. Psychol. Rev.* 28 162–178. 10.1016/j.cpr.2007.04.006 17618024

[B29] Nock M. K. Favazza A. R. (2009). “Nonsuicidal self-injury: definition and classification,” in *Understanding Nonsuicidal Self-Injury: Origins, Assessment, and Treatment*, ed. Nock M. K. (Washington, DC: American Psychological Association), 9–18. 10.1037/11875-001

[B30] Peng Y. S. Hu K. Wang Y. L. (2017). Age of separating from parents and parent-child attachment in left-behind children. *Chin. J. Clin. Psychol.* 25 731–734.

[B31] Prinstein M. J. (2008). Introduction to the special section on suicide and nonsuicidal self-injury: a review of unique challenges an important directions for self-injury science. *J. Consult. Clin. Psychol.* 76 1–8. 10.1037/0022-006X.76.1.1 18229976

[B32] Rawatlal N. Kliewer W. Pillay B. J. (2015). Adolescent attachment, family functioning and depressive symptoms. *S. Afr. J. Psychiatr.* 21:6 10.4102/sajpsychiatry.v21i3.672

[B33] Selby E. A. Bender T. W. Gordon K. H. Nock M. K. Joiner T. E. (2012). Non-suicidal self-injury (NSSI) disorder: a preliminary study. *Personal. Disord.* 3 167–175. 10.1037/a0024405 22452757

[B34] Su S. Li X. Lin D. Xu X. Zhu M. (2013). Psychological adjustment among left-behind children in rural China: the role of parental migration and parent-child communication. *Child Care Health Dev.* 39 162–170. 10.1111/j.1365-2214.2012.01400.x 22708901

[B35] Sümer N. Harma M. (2015). Parental attachment anxiety and avoidance predicting child’s anxiety and academic efficacy in middle childhood. *Psihologijske Teme* 24 113–134.

[B36] Sun L. X. Lin X. L. (2018). From “severe father and kind mother” to “kind mother and severe father”: parental differences in children’s strict discipline and their causes. *Educ. Res. Mon.* 8 55–62.

[B37] Wang Y. H. Chen J. Sun Y. Hu C. L. Tao F. B. (2013). Relationship between parent-child attachment and deliberate self-harm behaviors in middle school students. *Chin. J. Child Health Care* 21 1239–1242.

[B38] Wang Y. H. Zhang W. Peng J. X. Mo B. R. Xiong S. (2009). The relations of attachment, self -concept and deliberate self -harm in college students. *Psychol. Explor.* 29 56–61. 10.4040/jkan.2012.42.1.1 22410596

[B39] Wang Y. L. Yao Z. H. Jiang J. W. (2016). Parent-child attachment and emotion regulation ability of rural left-behind children: duration separated from parents as moderator. *Chin. J. Clin. Psychol.* 24 550–553.

[B40] Wang Y. L. Yuan Y. Zhang J. X. (2017). Negative emotion and self-injury in left-behind adolescents: moderating effect of family functioning and emotion expression. *Chin. J. Clin. Psychol.* 25 75–79.

[B41] Wu W. H. (2016). *Left-Behind Children’s Parent-Child Attachment Relations with Self-Injury Behavior: the Role of Social Self-Efficacy and Emotion Regulation Ability*. Changsha: Hunan Normal University.

[B42] Xu Y. Ma L. (2013). The characteristics and causes of self-harm behavior of left-behind adolescents in rural areas – based on the investigation in the city of Macheng in Hubei province. *J. South Central Univ. Natly.* 33 90–96.

[B43] Xu Z. W. Su H. Wu J. L. Chang W. W. Sun Y. H. (2010). Self-injurious behavior among rural stay-at-home middle school students and its relationship with internal and external locus of control. *Chin. J. Public Health* 26 868–869.

[B44] Yildiz M. A. (2016). Serial multiple mediation of general belongingness and life satisfaction in the relationship between attachment and loneliness in adolescents. *Educ. Sci. Theory Pract.* 16 553–578. 10.12738/estp.2016.2.0380

[B45] Zhao F. Yu G. (2016). Parental migration and rural left-behind children’s mental health in China: a meta-analysis based on mental health test. *J. Child Fam. Stud.* 25 3462–3472. 10.1007/s10826-016-0517-3

[B46] Zimmermann P. (1999). Structure and functions of internal working models of attachment and their role for emotion regulation. *Attach. Hum. Dev.* 1 291–306. 10.1080/14616739900134161 11708228

